# Adjuvant Antibacterial Effects of *Mouriri Elliptica* Against Clinical Multidrug Resistant Gram‐Negative Bacterial Strains

**DOI:** 10.1002/mbo3.70392

**Published:** 2026-09-09

**Authors:** Talita Vilalva Freire, Ana Carolina de Freitas Marques, Valéria dos Santos Gonçalves, Iluska Senna Bonfá Moslaves, Mônica Cristina Toffoli‐Kadri, Arthur Feliciano Camargo da Silva, Martha Janete Giz, Patrícia de Oliveira Figueiredo, Ana Camila Micheletti, Nídia Cristiane Yoshida

**Affiliations:** ^1^ Federal University of Mato Grosso do Sul, Laboratory of Research on Bioactive Natural Products (PRONABio), Institute of Chemistry (INQUI) Campo Grande Mato Grosso do Sul Brazil; ^2^ Federal University of Mato Grosso do Sul, Institute of Biology (INBIO) Campo Grande Mato Grosso do Sul Brazil; ^3^ Federal University of Mato Grosso do Sul, Faculty of Pharmaceutical Sciences (FACFAN) Campo Grande Mato Grosso do Sul Brazil; ^4^ Federal University of Mato Grosso do Sul, Laboratory of Electrochemistry of Advanced Materials (LEMA), Institute of Chemistry (INQUI) Campo Grande Mato Grosso do Sul Brazil

**Keywords:** *Acinetobacter baumannii*, antibiofilm, antimicrobial synergy, flavonoid, *Klebsiella pneumoniae*, natural products, One Health

## Abstract

Bacterial resistance to antibiotics represents a critical global health challenge, demanding urgent alternative strategies to manage infections caused by multidrug‐resistant (MDR) pathogens such as *Acinetobacter baumannii*, *Staphylococcus aureus*, *Escherichia coli*, and *Klebsiella pneumoniae*. A promising approach to address this problem involves the use of combination therapies, in which plant‐derived natural products represent an important reservoir of bioactive compounds with the potential to act as effective adjuvants, restoring or enhancing antibiotic efficacy. In this study, the ethanolic extract of *Mouriri elliptica* (EME) leaves was investigated for its antimicrobial and synergistic potential. EME showed moderate inhibitory activity against both standard and MDR strains, with the most significant effect against *A. baumannii* (MIC 78.1–312.5 µg/mL). Synergistic interactions were observed with conventional antibiotics, particularly ciprofloxacin against *K. pneumoniae*, reducing MIC values up to fifteen‐fold, and with ampicillin against *E. coli* and *S. aureus*. The extract also inhibited biofilm formation, with 68% inhibition for *E. coli* at 500 µg/mL and 72.04% for *S. aureus* at 250 µg/mL, with atomic force microscopy images corroborating these findings. LC‐MS profiling identified 41 compounds, including chlorogenic acid derivatives, flavonoids, ellagic acid derivatives, and triterpenes. Acute toxicity evaluation in mice at 2000 mg/kg revealed no adverse effects. Collectively, these findings support the potential of *M. elliptica* as a natural antibacterial adjuvant for enhancing the effectiveness of selected antibiotics against MDR pathogens, warranting further chemical, mechanistic, and in vivo investigation.

## Graphical abstract

The ethanolic leaf extract of *Mouriri elliptica* exhibited antibacterial and antibiofilm activities against multidrug‐resistant clinical isolates and enhanced the activity of ampicillin. Its diverse chemical profile and low acute toxicity support this Cerrado species as a promising source of natural antibacterial adjuvants.

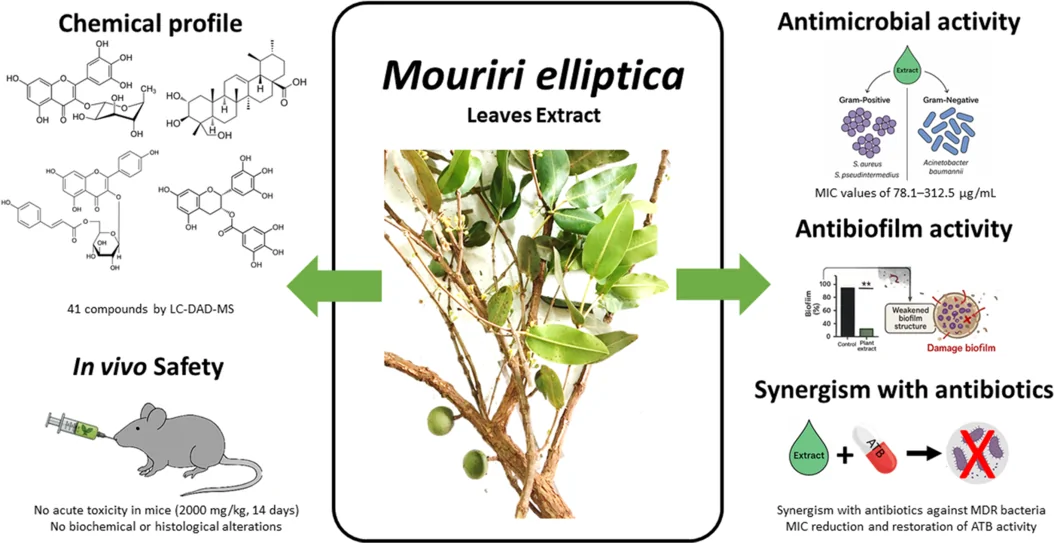

**Abbreviations**
AFM: atomic force microscopy
ALT: alanine aminotransferase
AST: aspartate aminotransferase
CEUA: Comissão de Ética no Uso de Animais (Ethics Committee on Animal Use)
CONCEA: Conselho Nacional de Controle de Experimentação Animal (Brazilian National Council for the Control of Animal Experimentation)
EME: ethanol extract of *Mouriri elliptica* leaves
H&E: hematoxylin–eosin
HCT: hematocrit
HGB: hemoglobin
MCH: mean corpuscular hemoglobin
MCHC: mean corpuscular hemoglobin concentration
MCV: mean corpuscular volume
OECD: Organization for Economic Co‐operation and Development
PLT: platelets
RBC: red blood cells
ROW: relative organ weight
SEM: standard error of the mean
WBC: white blood cells
WP/RP: white/red pulp.

## 1 Introduction

Multidrug resistance in bacteria is a significant and growing concern in infectious diseases and public health. MDR arises when bacteria develop resistance to multiple classes of antibiotics, thereby limiting the effectiveness of conventional therapies. This phenomenon occurs in both Gram‐positive and Gram‐negative bacteria and represents a serious threat to human health. Resistance may be either intrinsic or acquired, and drug‐resistant bacteria can disseminate among humans and animals through diverse routes, including food and the environment (Mallari et al. 2025). In the context of globalization, this spread is further exacerbated by international trade, travel, and human and animal migration (Coque et al. 2023).

Multidrug‐resistant (MDR) bacteria frequently evade conventional antibiotic therapies. A classification of 12 bacterial families, ranked by the urgency for new antibiotics, has been recognized as a serious threat to hospital environments and public health. This list includes MDR microorganisms associated with common infections, with particular emphasis on Gram‐negative bacteria resistant to multiple antibiotics, such as *Acinetobacter baumannii* and members of the Enterobacteriaceae family, as highlighted by the World Health Organization in 2024 (WHO Bacterial Priority Pathogens List 2024). The prevailing profile of antibiotic‐resistant bacteria in Brazilian hospitals included: carbapenem‐resistant *Acinetobacter* spp., oxacillin‐resistant coagulase‐negative staphylococci (ScoN), carbapenem‐resistant *Pseudomonas aeruginosa*, cephalosporin‐resistant *Escherichia coli* (both carbapenem‐sensitive and carbapenem‐resistant isolates), cephalosporin‐resistant *Enterobacter* spp., vancomycin‐resistant *Enterococcus* spp., cephalosporin‐resistant *Klebsiella pneumoniae* (both carbapenem‐sensitive and carbapenem‐resistant isolates), and oxacillin‐resistant *Staphylococcus aureus*, as recently reported by Brazilian Health Regulatory Agency (ANVISA Agência Nacional de Vigilância Sanitária 2020).

Overall, the rise of MDR bacteria represents a serious global health challenge that demands a multifaceted response, including enhanced surveillance, effective infection control measures, prudent antibiotic use, and the development of novel therapeutic strategies. Antimicrobial resistance is a complex phenomenon, driven by intricate interactions among diverse microbial populations that affect the health of humans, animals, and the environment. Addressing this issue requires a coordinated, multi‐sectoral approach, such as the One Health strategy, which integrates human, animal, and environmental health (Ferraz 2024). Bacteria and their resistance genes can readily circulate among these interconnected domains, and bacterial adaptation to antimicrobial use or other selection pressures in one domain is reflected across the others. Conversely, interventions to contain antimicrobial resistance in one sector can positively impact the others, underscoring the interconnected nature of this global problem.

Given these challenges, the search for new plant‐derived antibiotic agents has become imperative, with emphasis on the acquisition, mechanistic understanding (structure–activity relationships, SAR), and structural optimization of natural products. Research on the pharmacological potential of pure compounds as well as metabolite‐rich fractions (plant extracts and/or essential oils) has demonstrated antimicrobial activity against MDR human pathogens from a wide range of plant species (Subramani et al. 2017). In this context, the Brazilian Cerrado, a vast tropical savanna biome located primarily in central Brazil and comprising a diverse mosaic of grasslands, savannas, and woodlands, is recognized as a global biodiversity hotspot with remarkable plant diversity (Durigan 2025). Its rich flora represents a valuable source of bioactive natural products with potential antimicrobial and antibiotic‐adjuvant activities (de O. Ribeiro et al. 2018; De Jesus et al. 2020; Swain et al. 2025).

Plant‐derived secondary metabolites with antimicrobial action are structurally diverse. The most frequently reported bioactive compounds against microorganisms include quinones, phenols, tannins, terpenes, lignans, and glucosinolates, which act either individually or synergistically (as crude extracts, essential oils, or semi‐purified fractions) through distinct mechanisms that disrupt microbial resistance (Chandra et al. 2017). Among these classes, flavonoids, widely distributed phytoconstituents across plant tissues of Angiosperms, have been extensively reported to exhibit antimicrobial, antiviral, antiallergic, and anti‐inflammatory activities (Yuan et al. 2021; Bylka et al. 2004).

Species of the genus *Mouriri*, comprising approximately 87 species (Goldenberg 2015), have few reports of chemical or pharmacological studies. *Mouriri elliptica* (Mart.) (Melastomataceae), a native plant of the Brazilian Cerrado biome, represents a potential resource for human use. Its fruits are highly nutritious and rich in antioxidant compounds, and can be consumed fresh or processed into jams (Rufino et al. 2010). The leaves and bark of *M. elliptica* have traditionally been employed in the treatment of gastric ulcers and gastritis and may also exert antimicrobial effects (Moleiro et al. 2009).

Of the 87 recognized species, only *M. pusa* and *M. elliptica* have been chemically investigated to date, with reports describing the predominance of phenolic compounds, particularly flavonoids and their glycosylated derivatives, as well as ellagic acid, quinic acid, and gallic acid derivatives (Santos 2008; Vasconcelos et al. 2010a).

Despite these preliminary reports, the chemical composition of *M. elliptica* has not yet been comprehensively characterized, and its antibacterial, antibiofilm, and antibiotic‐adjuvant effects remain unexplored. Therefore, the present study aimed to characterize the chemical composition of the ethanolic extract of *M. elliptica* leaves (EME) and evaluate its antibacterial and antibiofilm activities against MDR bacteria isolated from human and veterinary clinical samples, as well as its interactions with conventional antibiotics. The acute in vivo toxicity of the extract was also assessed. The investigation of this underexplored species, native to the Brazilian Cerrado, highlights the potential of this biome's plant resources as sources of novel antibacterial adjuvants and contributes to their scientific valorization and conservation.

## 2 Results

### 2.1 Chemical Composition of *Mouriri elliptica*

**Figure 1:**
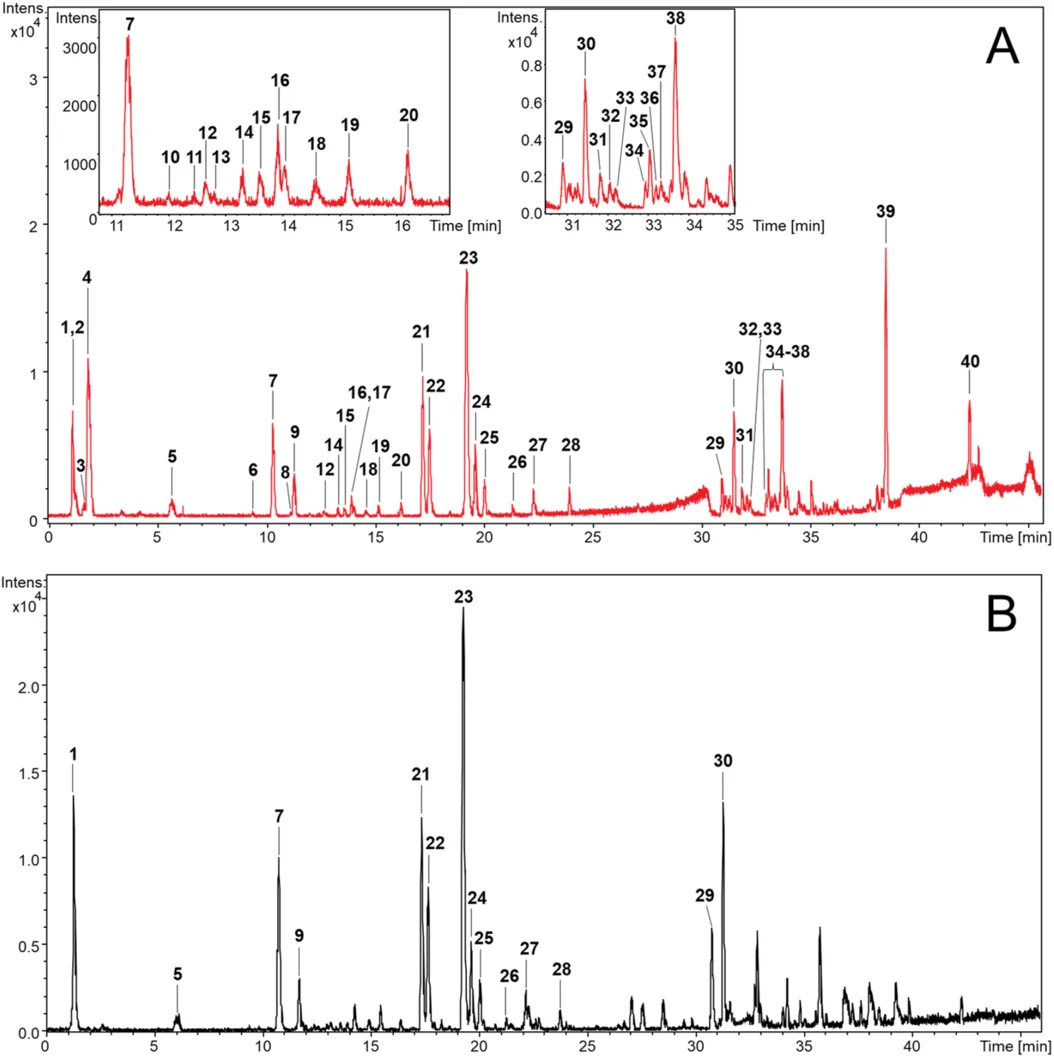
Base peak chromatograms (BPC) of the ethanol extract from leaves of *M. elliptica* (EME), obtained through HPLC‐UV‐MS/MS analysis. Peaks are numbered, corresponding to Table 1 compounds annotation (A: positive ionization mode; B: negative ionization mode).

The analysis of EME (Figure 1, Table 1) revealed diverse classes of compounds. Among these, saccharides were identified by two distinct peaks with retention times of 1.1 and 1.3 min ([M+Na]^+^ adducts of C_6_H_12_O_6_ and C_12_H_22_O_11,_ respectively). Additionally, a possible chlorogenic acid derivative was observed, represented by a singular peak at 12.0 min (*m/z* 609.1208), with a fragment of *m/z* 355.1029 (C_16_H_19_O_9_ [M + H]^+^; err −1.5 ppm) (Wang 2017).

**Table 1:** Tentative annotated compounds of *M. elliptica* leaves extract (EME) (* marker highlights *Mouriri* genus or *M. elliptica* isolation report; † marker corresponds to an *in‐source* fragment ion).

Peak number	Experimental MS (*m/z*)	Retention time (min)	Molecular formula [adduct type]	Error (ppm)	MSE fragments (relative intensity)	UV (nm)	Tentative identification	Annotation level of confidence	Melastomataceae previous annotated compound (References)
1	203.0532	1.1	C_6_H_12_O_6_ [M+Na]^+^	−2.8	—	N.D.	Glucose (glucopyranose); Frutose; or Galactose (galactopyranose)	3	Zakaria et al. 2016
2	365.1048	1.3	C_12_H_22_O_11_ [M+Na]^+^	1.7	—	N.D.	4‐O‐α‐d‐Glucopyranosyl‐d‐glucopyranose; Maltose; Sucrose or Trehalose	3	—
3	231.0830	1.6	C_5_H_15_N_2_O_8_ [M + H]^+^ C_8_H_16_O_6_ [M+Na]^+^	−3.0 4.0	—	N.D.	Unknown	4	—
4	231.0834	1.9	C_5_H_15_N_2_O_8_ [M + H]^+^ C_8_H_16_O_6_ [M+Na]^+^	−5.0 2.0	—	210	Unknown	4	—
5	611.1404	5.7	C_30_H_27_O_14_ [M + H]^+^	−1.5	—	210	Helichrysoside or Prodelphinidin B3	3	Halim et al. 2017; Rezende et al. 2019
6	595.1456 (88.80)	9.4	C_30_H_27_O_13_ [M + H]^+^	−1.7	—	210	Tiliroside	3	Rezende et al. 2019
7	307.0799	10.3	C_15_H_15_O_7_ [M + H]^+^	4.4	—	210; 270	Gallocatechin or Epigallocatechin	3	
8	899.1952	11.1	C_22_H_47_N_2_O_35_ [M + H]^+^ C_50_H_36_O_15_ [M+Na]^+^	0.2 −0.7	—	210	Unknown	4	—
9	595.1435	11.3	C_30_H_27_O_13_ [M + H]^+^	1.8	—	280	Tiliroside	3	Rezende et al. 2019
10	609.1208 (39.5)	12.0	C_28_H_26_O_14_ [M+Na]^+^ C_41_H_18_N_2_O_3_ [M+Na]^+^	1.2 0.3	355.1029^†^ (100)	210	Unknown	4	—
11	883.1938	12.5	C_41_H_39_O_22_ [M + H]^+^ C_42_H_35_N_4_O_18_ [M + H]^+^	−1.2 0.3	—	210	Unknown	4	—
12	889.2006	12.6	C_52_H_35_O_15_ [M + H]^+^ C_52_H_34_O_15_ [M+Na]^+^ C_40_H_39_N_2_O_22_ [M + H]^+^	−3.9 −3.3 −1.8	—	210	Unknown	4	—
13	954.1169	12.8	C_23_H_40_NO_39_ [M + H]^+^	0.3	—	210	Unknown	4	—
14	611.1388	13.3	C_30_H_27_O_14_ [M + H]^+^	1.1	—	210	Helichrysoside or Prodelphinidin B3	3	Halim et al. 2017; Rezende et al. 2019
15	579.1493	13.6	C_30_H_27_O_12_ [M + H]^+^	0.7	—	210; 281	Procyanidin B2	3	Kumar et al. 2021
16	291.0892	13.9	C_15_H_15_O_6_ [M + H]^+^	−9.9	—	210; 277	Catechin; Epicatechin	3	Wang et al. 2017
17	883.2058	14.0	C_28_H_43_N_4_O_28_ [M + H]^+^ C_43_H_40_O_19_ [M+Na]^+^	0.0 −0.2	—	210; 280	Unknown	4	—
18	459.0910 (56.06)	14.6	C_22_H_19_O_11_ [M + H]^+^	2.5	327.2026 (100)	212; 277	Epigallocatechin gallate	3	Gordon et al. 2011
19	409.1850 (47.97)	15.2	C_25_H_26_N_2_O_2_ [M+Na]^+^ C_21_H_29_O_8_ [M + H]^+^ C_19_H_30_O_8_ [M+Na]^+^	8.8 1.6 −4.3	207.1363^†^ (100)	212; 277	Unknown	4	—
20	595.1428	16.2	C_30_H_27_O_13_ [M + H]^+^	3.1	—	212; 277	Tiliroside	3	Rezende et al. 2019
21	481.0989 (55.38) 479.0845	17.2	C_21_H_21_O_13_ [M + H]^+^ C_21_H_19_O_13_ [M + H]^‐^	−2.5 −3.0	319.0453^†^ (100) 316.0236 (100)	355	Myricetin 3‐O‐glucoside; Myricetin 3‐O‐galactoside or 2‐[4‐(β‐d‐Arabinopyranosyloxy)−3‐hydroxyphenyl]−3,5,7‐trihydroxy‐4H‐1‐benzopyran‐4‐one	3	Vasconcelos et al. 2010
22	481.0980 (40.89)	17.5	C_21_H_21_O_13_ [M + H]^+^	−0.7	319.0441^†^ (100)	355	Myricetin 3‐O‐glucoside; Myricetin 3‐O‐galactoside or 2‐[4‐(β‐d‐Arabinopyranosyloxy)−3‐hydroxyphenyl]−3,5,7‐trihydroxy‐4H‐1‐benzopyran‐4‐one	3	Vasconcelos et al. 2010
23	465.1026 (20.11) 463.0895	19.2	C_21_H_21_O_12_ [M + H]^+^ C_21_H_19_O_12_ [M + H]^‐^	0.4 −2.9	319.0448^†^, 273.0342 (100) 316.0237 (100)	355	Myricitrin	3	Vasconcelos et al., 2010
24	487.0840 (35.41)	19.6	C_21_H_20_O_12_ [M+Na]^+^	1.6	465.1022^†^ (42.94), 303.0498^†^ (100)	355	Quercetin 3‐*O*‐glucoside, Hyperoside; Isoquercitrin, Quercetin 7‐*O*‐glucoside, 6‐Hydroxyluteolin 7‐glucoside, 7‐(β‐d‐Glucopyranosyloxy)−3,5,6‐trihydroxy‐2‐(4‐hydroxyphenyl)−4H‐1‐benzopyran‐4‐one, Cyanidol 3‐galactoside or 2‐(3,4‐Dihydroxyphenyl)−6‐(β‐d‐glucopyranosyloxy)−5,7‐dihydroxy‐4H‐1‐benzopyran‐4‐one	3	Vasconcelos et al. 2010
25	487.0843 (33.20)	20.0	C_21_H_20_O_12_ [M+Na]^+^	0.9	465.1020^†^ (33.58), 303.0496^†^ (100)	355	Quercetin 3‐*O*‐glucoside, Hyperoside; Isoquercitrin, Quercetin 7‐*O*‐glucoside, 6‐Hydroxyluteolin 7‐glucoside, 7‐(β‐d‐Glucopyranosyloxy)−3,5,6‐trihydroxy‐2‐(4‐hydroxyphenyl)−4H‐1‐benzopyran‐4‐one, Cyanidol 3‐galactoside or 2‐(3,4‐Dihydroxyphenyl)−6‐(β‐d‐glucopyranosyloxy)−5,7‐dihydroxy‐4H‐1‐benzopyran‐4‐one	3	Vasconcelos et al. 2010
26	471.0904 (61.35)	21.3	C_21_H_20_O_11_ [M+Na]^+^	−1.2	287.0550^†^ (100)	217	Astragalin, Plantaginin, Kaempferol 3‐O‐galactoside, 6‐(β‐d‐Glucopyranosyloxy)−5,7‐dihydroxy‐2‐(4‐hydroxyphenyl)−4H‐1‐benzopyran‐4‐one; 5,7‐Dihydroxy‐2‐(4‐hydroxyphenyl)−6‐methoxy‐3‐(β‐d‐xylopyranosyloxy)−4H‐1‐benzopyran‐4‐one; 2‐[4‐(β‐d‐Glucopyranosyloxy)phenyl]−5,6,7‐trihydroxy‐4H‐1‐benzopyran‐4‐one or 6‐(β‐d‐Galactopyranosyloxy)−5,7‐dihydroxy‐2‐(4‐hydroxyphenyl)−4H‐1‐benzopyran‐4‐one	3	Vasconcelos et al. 2010
27	471.0972 (15.38)	22.3	C_16_H_23_O_16_ [M + H]^+^	1.9	303.0471^†^ (100)	355	Quercitrin, Luteolin 7‐O‐glucoside, Isoorientin, Orientin, Cyanidin 3‐O‐β‐d‐galactoside, Luteolin‐7‐O‐galactoside, 6‐(β‐d‐Glucopyranosyloxy)−5,7‐dihydroxy‐2‐(4‐hydroxyphenyl)−4H‐1‐benzopyran‐4‐one; 5,7‐Dihydroxy‐2‐(4‐hydroxyphenyl)−6‐methoxy‐3‐(β‐d‐xylopyranosyloxy)−4H‐1‐benzopyran‐4‐one; 2‐[4‐(β‐d‐Glucopyranosyloxy)phenyl]−5,6,7‐trihydroxy‐4H‐1‐benzopyran‐4‐one or 6‐(β‐d‐Galactopyranosyloxy)−5,7‐dihydroxy‐2‐(4‐hydroxyphenyl)−4H‐1‐benzopyran‐4‐one	3	Vasconcelos et al. 2010
28	329.0278	23.9	C_16_H_9_O_8_ [M + H]^+^	4.3	—	353	Ellagic acid derivative: 56470‐19‐0	3	Cho et al. 2011
29	329.0292	30.9	C_16_H_9_O_8_ [M + H]^+^	0.0	—	220; 370	Ellagic acid derivative: 56470‐19‐0	3	Cho et al. 2011
30	489.3567	31.5	C_30_H_49_O_5_ [M + H]^+^	1.6	425.3485 (17.42), 273.1839 (15.73), 243.2145 (13.76), 229.1742 (20.79), 217.1536 (17.98), 213.1634 (17.98), 205.1585 (67.13), 203.1741 (100),	220	Asiatic acid or Arjunolic acid	3	Rezende et al. 2019
31	689.2968	31.8	C_37_H_46_O_11_ [M+Na]^+^ C_25_H_50_N_2_O_18_ [M+Na]^+^	−5.2 −2.5	—	221	Unknown	4	—
32	705.3344 (44.46)	32.1	C_33_H_53_O_16_ [M + H]^+^ C_34_H_49_N_4_O_12_ [M + H]^+^	−2.3 −0.4	—	221; 277	Unknown	4	—
33	777.3534	32.2	C_36_H_57_O_18_ [M + H]^+^	0.7	—	221; 277	Unknown	4	—
34	453.3379	32.9	C_30_H_45_O_3_ [M + H]^+^ C_28_H_46_O_3_ [M+Na]^+^	−3.4 −8.8	—	221	Unknown Triterpene	4	—
35	453.3342	33.0	C_30_H_45_O_3_ [M + H^]+^ C_28_H_46_O_3_ [M+Na]^+^	4.7 −0.6	—	221	Unknown Triterpene	4	—
36	527.3332 (47.31)	33.2	C_30_H_48_O_6_ [M+Na]^+^	2.1	469.3322^†^ (100)	221	Terminolic acid or Myrianthic acid	3	Zhang et al. 2003; Kenfack et al. 2018
37	245.0809 (67.90)	33.3	C_14_H_13_O_4_ [M + H]^+^	−0.2	177.0541^†^ (100)	277	Unknown	4	—
38	343.2959	33.6	C_22_H_40_O [M+Na]^+^	3.5	—	221; 277	Unknown	4	—
39	457.2752	38.4	C_22_H_42_O_8_ [M+Na]^+^ C_19_H_41_N_2_O_10_ [M + H]^+^	4.4 −2.3	369.2098 (0.96); 313.1614 (3.87)	223	Unknown	4	—
40	439.3568	42.2	C_28_H_48_O_2_ [M+Na]^+^	−4.9	—	223	(3β,4α,5α)−14‐Methyl‐9,19‐cyclocholestane‐3,4‐diol	3	Leite et al. 2013
41	803.5429 (29.65)	44.2	C_45_H_75_N_2_O_10_ [M + H]^+^ C_48_H_76_O_8_ [M+Na]^+^	−1.6 0.4	—	223	Unknown	4	—

Flavonoids comprised a substantial portion of EME, with a total of 11 peaks detected. These peaks presented a retention time range from 17.2 to 22.3 min and included specific flavonoids such as catechin/epicatechin (rt = 13.9 min), and derivatives of quercetin (total of three peaks), kaempferol (one peak), and myricetin (total of three peaks), all in glycosylated forms. These aglycones were proposed to each peak according to observed *in‐source* MS (Coque et al. 2023) ions (quasi‐molecular ions [M + H]^+^): *m/z* 319 (C_15_H_11_O_8_) for myricetin, *m/z* 303 (C_15_H_11_O_7_) for quercetin, and *m/z* 287 (C_15_H_11_O_6_) for kaempferol (Cuyckens and Claeys 2004; Aziz et al. 2022). These findings align with earlier research reporting this metabolite class on the leaves of this species (Moleiro et al. 2009; Santos 2008; Girondi et al. 2017). Compounds **5**, **6**, **14** and **15** were also part of this category being tentatively annotated as Procyanidin B_2_ (C_30_H_27_O_12_ [M + H]^+^), Tiliroside (C_30_H_27_O_13_ [M + H]^+^), Prodelphinidin B_3_ or Helichrysoside (C_30_H_27_O_14_ [M + H]^+^); these flavonoid dimers or featuring C_6_‐C_3_ units are herein reported on *Mouriri* genus for the first time. Additionally, compounds **28** and **29** matched with di‐*O*‐methyl‐ellagic acid derivatives, presenting retention times of 23.9 and 30.9 min (C_16_H_9_O_8_ [M + H]^+^; err = 4.3 and 0.0, respectively).

**Figure 2:**
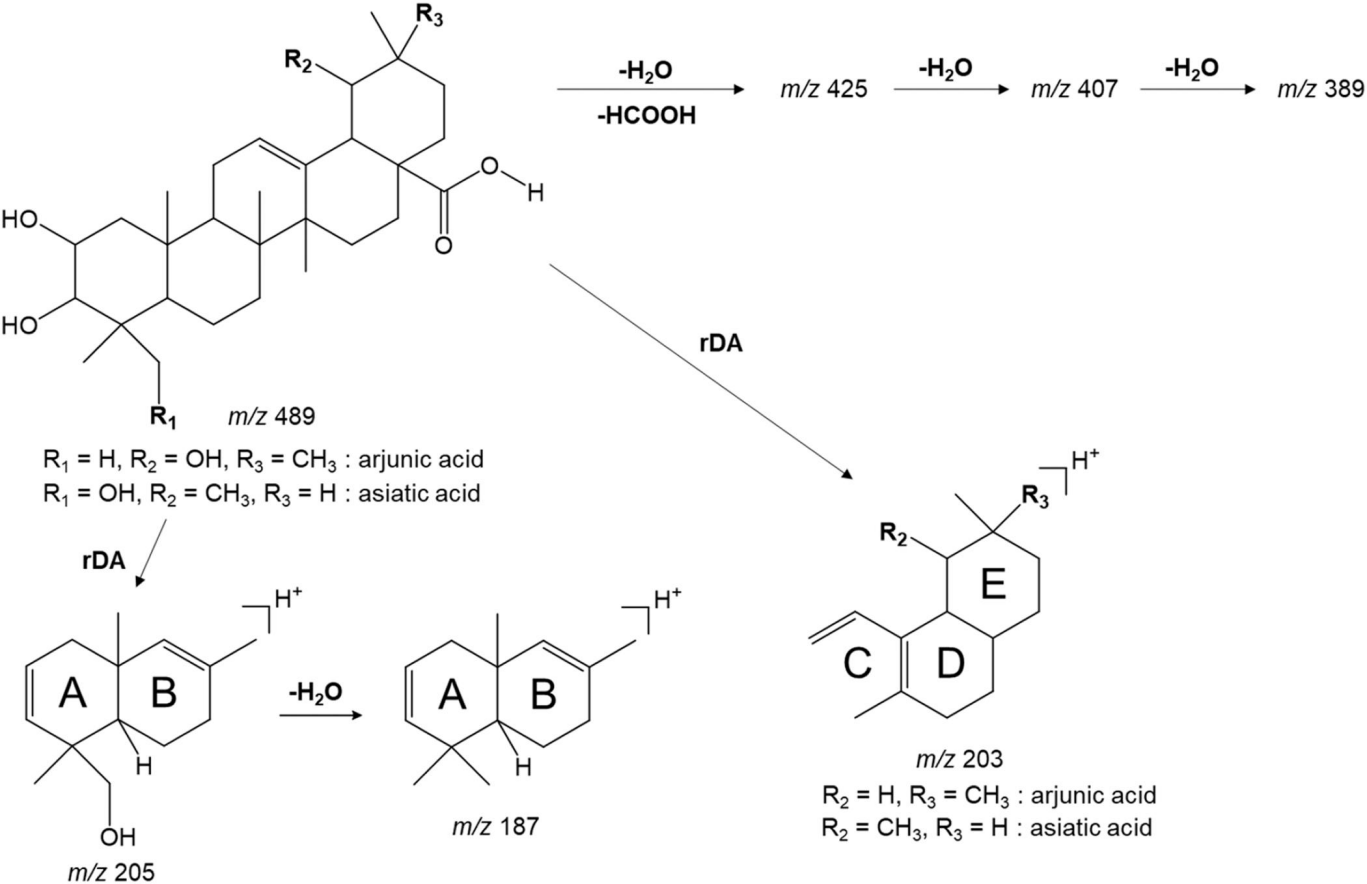
Proposed fragmentation of compound **30**, based on (Uddin et al. 2022). Eschweilenol C, an ellagic acid derivative previously reported on ethanolic extract of *M. elliptica* (Girondi et al. 2017), was not detected in positive nor negative ESI mode in the present study.

Furthermore, pentacyclic triterpenes, hitherto unknown within *Mouriri*, were identified (compounds **30** and **34**), represented by two peaks at retention times of 31.5 and 33.2 min. Mass spectra data of **30** presented a quasi‐molecular ion of *m/z* 489.3567, corresponding to the molecular formula C_30_H_49_O_5_ ([M + H]^+^; err 1.6 ppm). Key fragmentation pattern of this ion was observed at *m/z* 203.1741 (base peak), corresponding to a classic retro Diels‐Alder pathway (rDA) for both oleanane and ursane‐triterpenoids, as well as *m/z* 425.3485 ([M + H‐HCOOH‐H_2_O]^+^), 205.1585 (C_14_H_21_O^+^), and 187.1539 (C_14_H_19_^+^) (Figure 2). Despite the presence of additional fragments at *m/z* 273, *m/z* 243, *m/z* 229, *m/z* 217, *m/z* 213, *m/z* 173, and *m/z* 159, it is challenging to conclusively attribute this molecule identity. Although its potential correspondence to arjunic acid, a more strongly supported identification points towards asiatic acid. This determination is based on comparisons with *in‐house* Melastomataceae library and aligns with the characteristic fragmentation patterns of ursane‐type triterpenoids observed on ESI‐QTOFMS/MS, as reported by Uddin et al. (2022).

### 2.2 Antimicrobial Potential

According to (Wamba et al. 2018) and (Simões et al. 2009), the antibacterial activity of plant extracts is considered significant if the MIC values are below 100 µg/mL, moderate if 100 ≤ MICs ≤ 625 µg/mL, and weak if MICs > 625 µg/mL. On the other hand, the antimicrobial activity of an isolated phytochemical can be considered significant when MIC is below 10 µg/mL, moderate when 10 µg/mL < MIC < 100 µg/mL, and weak when MIC > 100 µg/mL (Mbaveng et al. 2015). The effect of an antimicrobial combination is considered synergistic when fractional inhibitory concentration index (FICI) is less than or equal to 0.5, additive when 0.5 < FICI ≤ 1, indifferent when 1 < FICI ≤ 4, and antagonistic when FICI > 4 (Silva et al. 2019).

For the antibiotics ampicillin and ciprofloxacin, the MIC values ranged from 3.125 to > 200 μg/mL. Therefore, the MIC results found against the tested bacteria for the action of EME alone indicated strong activity against the standard strain of *S. aureus* NEWP0023, with MIC < 78.1 µg/mL; moderate activity against multidrug‐resistant clinical strains of *S. aureus* and *S. pseudointermedius* with MIC = 156.25 µg/mL, against *Acinetobacter baumannii* with MIC = 312.5 µg/mL; and against the standard sensitive strain of *E. coli* and multidrug‐resistant clinical strains of *E. coli* B, *Staphylococcus* sp., and *Klebsiella pneumoniae* with MIC = 625 µg/mL; followed by weak activity against *E. coli* NEWP0018, β‐lactamase producer, and *E. coli* A with MIC = 1250 µg/mL (Table 2).

Although the EME alone showed moderate activity against almost all the tested strains, experiments were conducted to test the combination of the extract with antibiotics to which the bacteria had already shown resistance. This approach was applied to check on synergistic interactions resulting from these combinations, which has proven to be a very productive strategy when it comes to natural plant‐based products. The results of these assays are presented in Table 2.

**Table 2:** Minimum inhibitory concentration (MIC), fractional inhibitory concentration (FIC) and FIC indices (FICI) of the combination of antibiotic‐extracts against tested bacteria. EME: Ethanolic Extract from the Leaves of *Mouriri elliptica*. AMP: Ampicillin. CIP: Ciprofloxacin.

Bacteria	Sample/Combination	MIC of isolated sample (µg/mL)	MIC of combination (µg/mL)	FIC	FICI	Effect
*S. pseudointermedius*	EME + AMP				0.516	Additive
	EME	156.2	78.1	0.5		
	AMP	100	1.56	0.016		
*E. coli* NEWP0018	EME + AMP				0.09	Synergism
	EME	1250	39.1	0.03		
	AMP	400	25	0.06		
*E. coli* A	EME + AMP				0.53	Additive
	EME	1250	625	0.5		
	AMP	3.125	0.09	0.03		
*Staphylococcus* sp.	EME + AMP				0.75	Additive
	EME	625	156.25	0.25		
	AMP	3.125	1.56	0.5		
*S. aureus*	EME + AMP				0.5	Synergism
	EME	156.2	39.1	0.25		
	AMP	6.25	1.56	0.25		
*E. coli* B	EME + AMP				2	Indifferent
	ME	625	1250	2		
	AMP	100	0.09	0.0009		
*A. baumannii*	EME + CIP				0.625	Additive
	EME	312.5	39.1	0.125		
	CIP	25	12.5	0.5		
*K. pneumaniae*	EME + CIP				0.19	Synergism
	EME	625	39.1	0.0625		
	CIP	> 200	50	0.125		

*S. aureus* resistant to penicillin G, erythromycin, and clarithromycin; *Staphylococcus* sp., resistant to amoxicillin, doxycycline, clindamycin, penicillin, oxacillin, norfloxacin, cefoxitin, azithromycin (from veterinary source); *E. coli* resistant to clindamycin, penicillin, and oxacillin (Shiga toxin‐producing *E. coli* A, from veterinary source); *E. coli* resistant to azithromycin, ampicillin, doxycycline, clindamycin, penicillin, oxacillin, norfloxacin, and cefoxitin (*E. coli* B, from veterinary source); *S. pseudointermedius* resistant to amoxicillin + clavulanic acid, gentamicin, neomycin, azithromycin, cephalexin, cephalothin, streptomycin, marbofloxacin (from veterinary source); *A. baumannii* resistant to amikacin, ciprofloxacin, gentamicin, imipenem, levofloxacin, and meropenem and *K. pneumoniae* resistant to amoxicillin‐clavulanate, ampicillin, aztreonam, cefepime, cefoxitin, ceftriaxone, cefuroxime, ciprofloxacin, colistin, ertapenem, gentamicin, imipenem, levofloxacin, meropenem, piperacillin‐tazobactam, intermediate to tigecycline.

Synergistic effects were observed when the EME was combined with ampicillin, resulting in a fractional inhibitory concentration index (FICI) of 0.5 against clinical *S. aureus*, 0.19 against *K. pneumoniae*, and 0.09 against *E. coli* (NEWP0018). In these combinations, the lowest concentration of the extract resulted in a 4–fold reduction in the antibiotic MIC against *S. aureus* and *K. pneumoniae,* and 16‐fold reduction against *E. coli*. Notably, the extract contributed to the restoration of ampicillin and ciprofloxacin antibacterial activity against resistant strains.

### 2.3 Antibiofilm Activity

**Figure 3:**
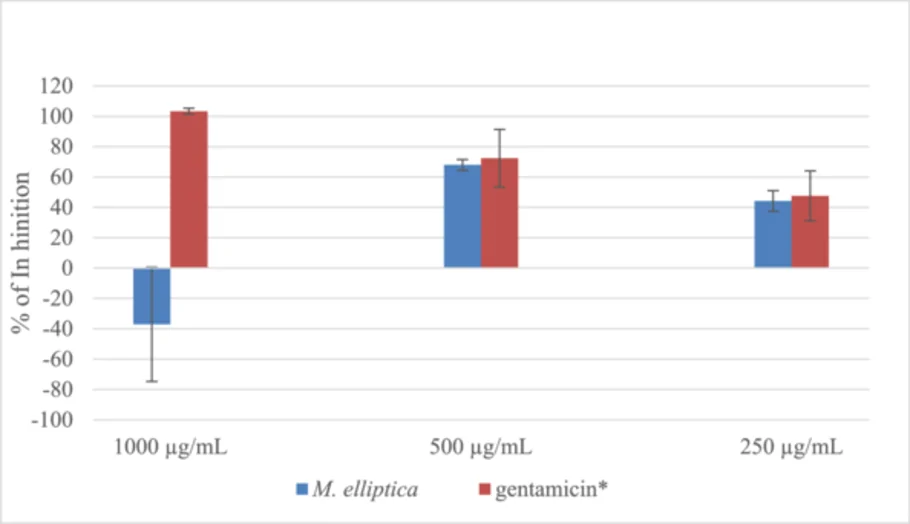
Biofilm formation by *Escherichia coli* NEWP0022 treated with Ethanolic Extract from the Leaves of *M. elliptica*. *Gentamicin was evaluated in a 100× lower concentration.

**Figure 4:**
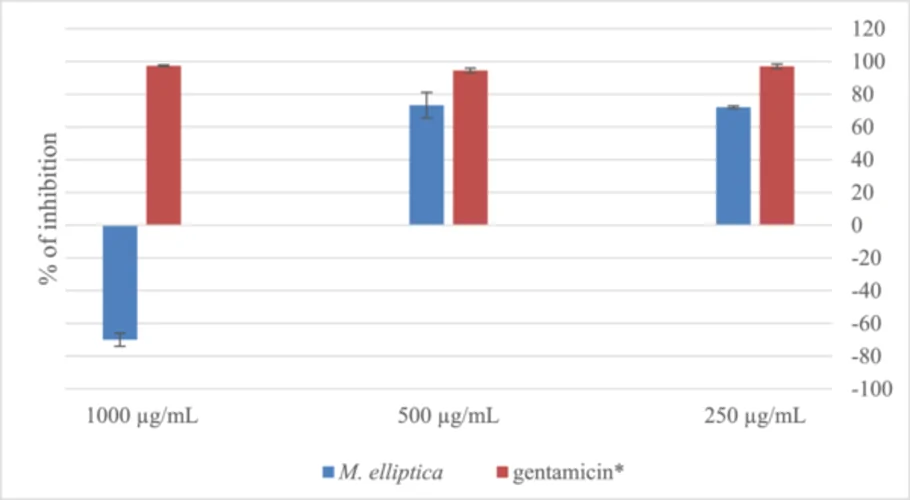
Biofilm formation by *Staphylococcus aureus* NEWP0023 treated with Ethanolic Extract from the Leaves of *M. elliptica*. *Gentamicin was evaluated in a 100× lower concentration.

Antibiofilm potential of the EME was also evaluated against two stand strains: *S. aureus* NEWP0023 (Gram‐positive) and. *E. coli* NEWP0022 (Gram‐negative), as biofilm formation is an important resistance strategy for bacterial cells. Results are summarized in Figures 3 and 4.

As illustrated in Figures 3 and 4, the EME exhibited a similar pattern of activity against both bacterial strains, promoting biofilm formation at the highest concentration (1000 µg/mL) while inhibiting its formation at lower concentrations. For both strains, the most pronounced inhibitory effect was observed at 500 µg/mL, resulting in a 73.3% reduction in *S. aureus* biofilm formation and a 68% reduction in *E. coli* biofilm formation. At 250 µg/mL, however, inhibition of *S. aureus* biofilm formation remained high (72.04%), whereas the inhibitory effect on *E. coli* decreased markedly to 44.2%.

**Figure 5:**
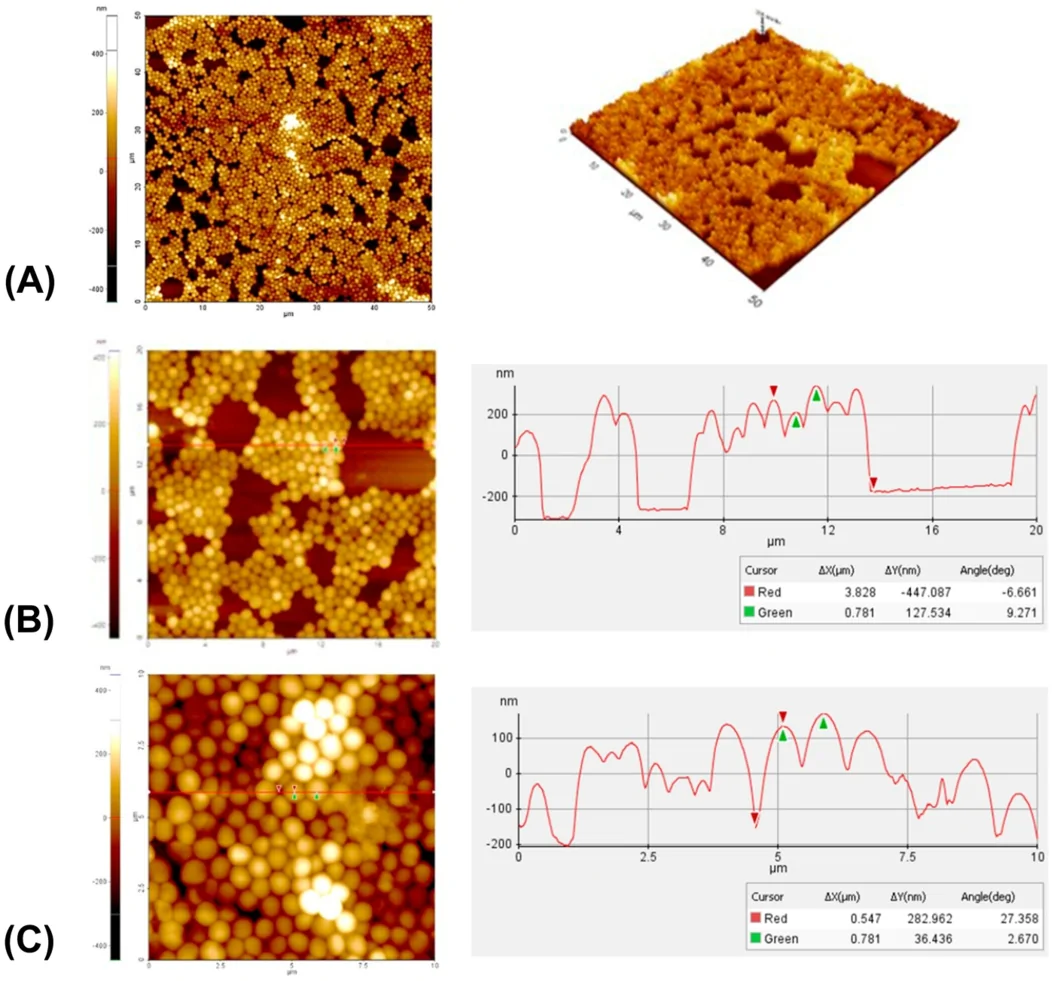
Representative AFM Topographic images of *S. aureus* biofilm structure: (A) 2D and 3D, 50 × 50 μm scan size, (B) 2D and histogram, 20 × 20 μm scan size, and (C) 10 × 10 μm scan size.

**Figure 6:**
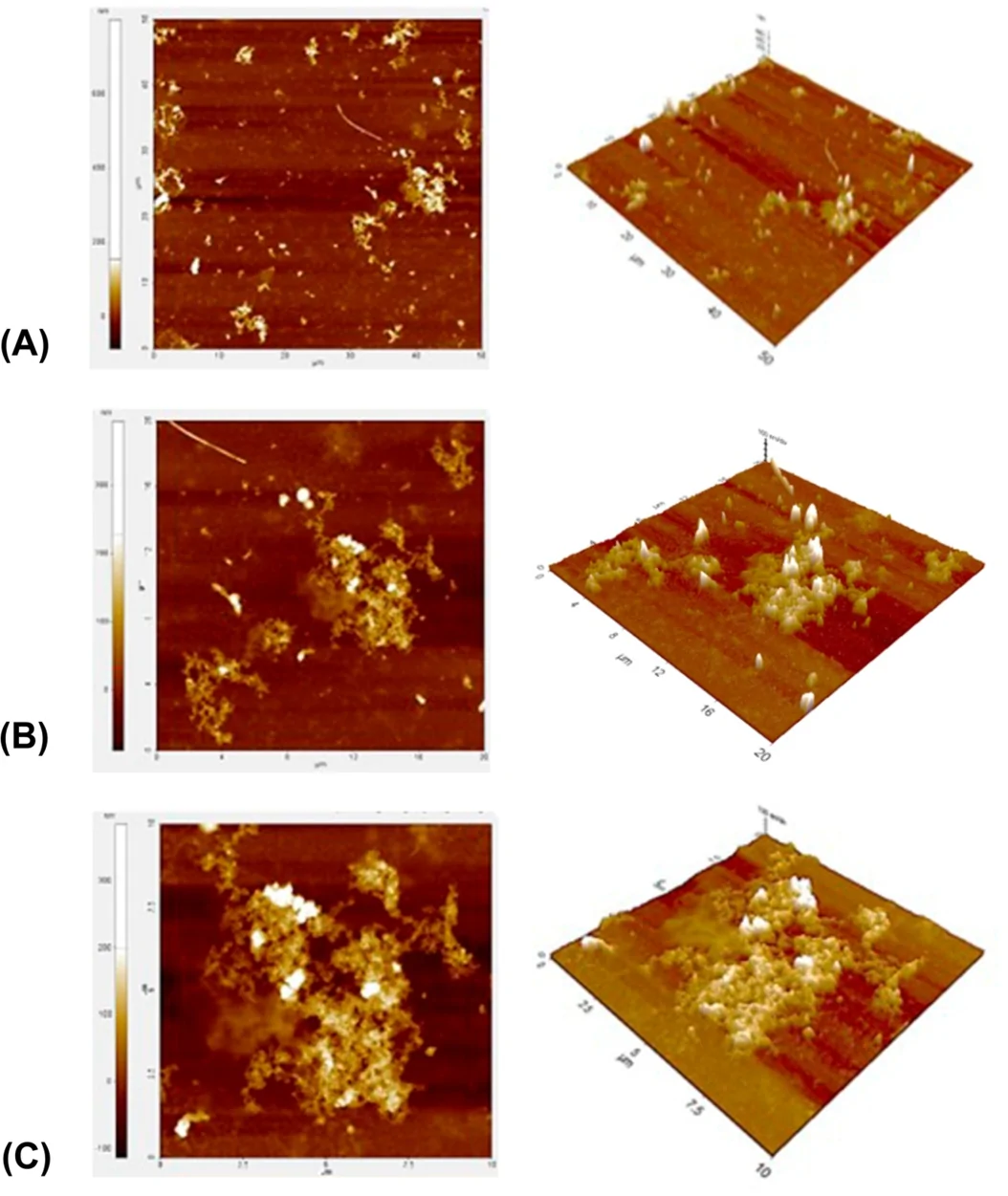
Topography (2D and 3D view) images of biofilm inhibition: scan sizes (A) 50 × 50 μm, (B) 20 × 20 μm, and (C) 10 × 10 μm.

The effects of EME on *S. aureus* biofilm formation were evaluated by atomic force microscopy (AFM). Figures 5 and 6 show representative topographic images of the untreated *S. aureus* culture (control) and the inhibition of film formation by EME at 250 µg/mL, respectively, at different scan sizes. In the untreated control, spherical bacterial cells were homogeneously distributed within a well‐organized biofilm structure (Figure 5A). The cells exhibited an approximate diameter of 780 nm and a height of 280 nm (Figure 5B,C), consistent with previous reports (Kao Godínez et al. 2025). In contrast, treatment with the extract markedly inhibited biofilm formation and caused substantial disruption of the biofilm architecture (Figure 6), demonstrating its pronounced antibiofilm effect.

### 2.4 In Vivo Toxicity Assay

**Figure 7:**
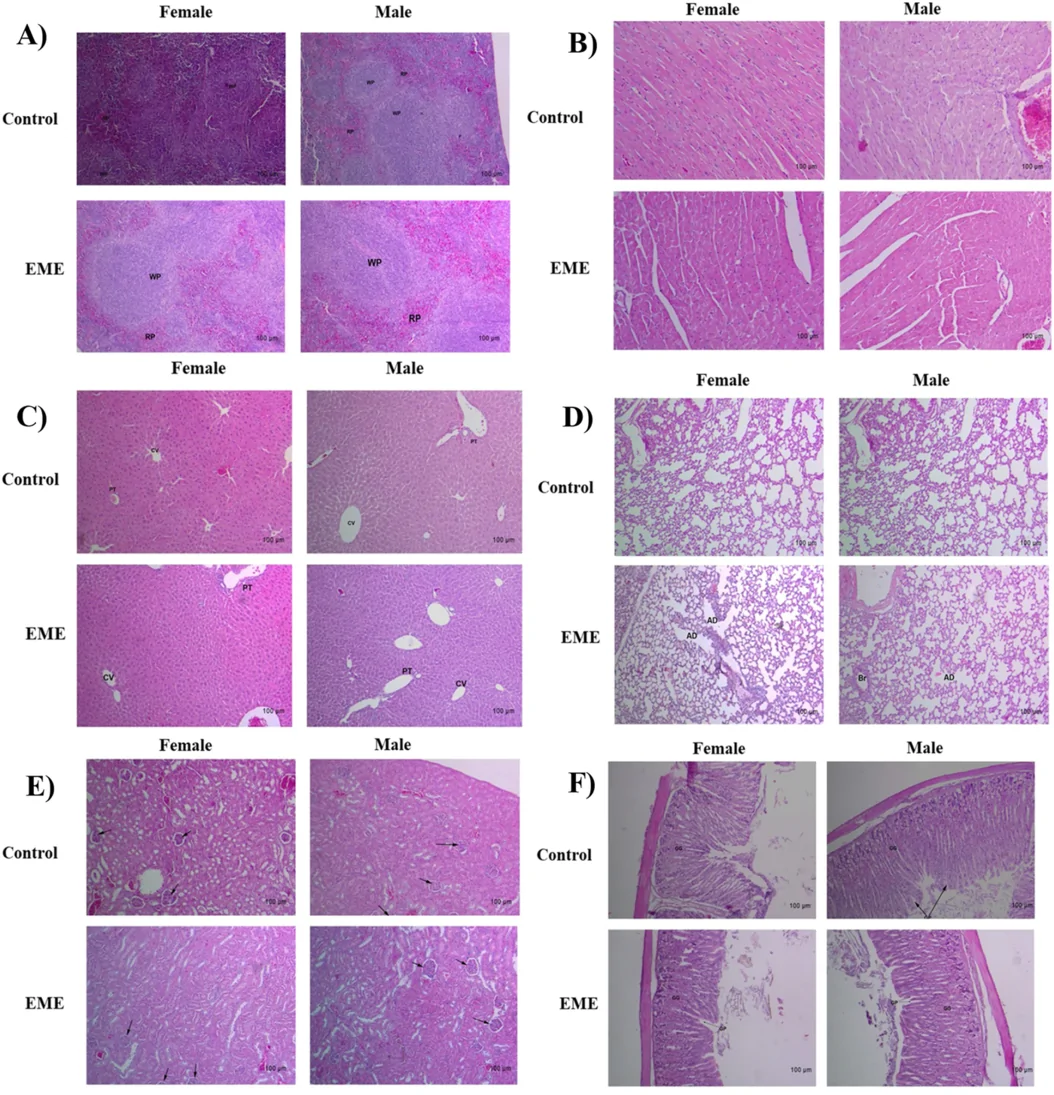
Histopathological analysis of the (A) spleen (WP, white pulp; RP, red pulp); (B) heart; (C) liver (CV = Centrolobular vein. PT = Portal triad); D) lungs (AD = Aleveolar duct. BR = Bronchiole); (E) kidneys; (F) stomach (GG = Gastric glands. GP = Pit gastric); in male and female mice after treatment with the ethanolic extract of *M*. *elliptica* leaves (EME), compared with the control group (water). Magnification: 100×. Hematoxylin–eosin (H&E) staining.

Acute toxicity was evaluated in vivo following treatment with EME. The assays included monitoring of body weight gain, relative organ weights, hematological and biochemical parameters, as well as histopathological analysis of the spleen, heart, liver, kidneys and stomach. No significant variations were observed between treated and control groups, with all organs displaying morphological features within normal parameters, indicating that EME was well tolerated and exhibited no evidence of acute systemic toxicity in the animal model (Figure 7, Supporting Information S1: Tables S1 and S2).

### 2.5 Hematological Parameters in the Acute Toxicity Assay

Hematological analysis showed that most parameters, including RBC, HGB, HCT, MCV, MCH, MCHC, and PLT, remained unchanged between control and treated groups in both sexes. In females, no significant variations were observed in any of the evaluated parameters. In males, treatment with EME resulted in a significant increase in WBC counts compared with controls (*p* < 0.05), while all other parameters remained within normal ranges. These data indicated that the extract did not cause hematological disturbances under the conditions of the acute toxicity assay (Supporting Information S1: Table S3).

### 2.6 Biochemical Parameters

Biochemical evaluation revealed that glucose, albumin, ALT, and AST levels did not differ significantly between control and treated groups in either sex. In contrast, bilirubin levels were significantly reduced in females (*p* < 0.05) and markedly decreased in males (*p* < 0.001) following treatment with EME. Overall, these findings indicate that the extract did not induce biochemical alterations suggestive of hepatic or systemic toxicity under the conditions of the acute toxicity assay (Supporting Information S1: Table S4).

## 3 Discussion

The ethanolic extract of *M. elliptica* leaves (EME) exhibited antibacterial activity against the reference strain *Staphylococcus aureus* NEWP0023 with a minimum inhibitory concentration (MIC) below 78.1 µg/mL. EME also showed moderate activity against clinical isolates of *S. pseudintermedius* and a resistant strain of *S. aureus*, with an MIC of 156.25 µg/mL for both strains. Importantly, several of the tested strains—*Staphylococcus sp*., *E. coli* (A and B), and *S. pseudointermedius*—were from animal origin, reinforcing the relevance of these findings within a One Health approach, since resistant bacteria from veterinary settings can disseminate to humans through direct contact, food chains, or the environment. Such pathogens not only represent a risk to human and animal health but also impose significant economic impacts on livestock production due to treatment failures, prolonged infections, and productivity losses.

EME exhibited moderate activity against multidrug‐resistant Gram‐negative bacteria, including *A. baumannii* (MIC 312.5 µg/mL) and *K. pneumoniae* (MIC 625 µg/mL). The reduced susceptibility of these pathogens may be associated with intrinsic resistance mechanisms and the structural complexity of their outer membrane, which contains lipopolysaccharides and transmembrane proteins, such as porins, that restrict the penetration of antimicrobial agents. Such features account for their persistence and limited susceptibility to conventional therapies, underscoring the urgent need for novel antimicrobial strategies (Silva et al. 2019; Oliveira et al. 2025; Dettori et al. 2023; Breijyeh et al. 2020).

Synergistic interactions were also observed between EME and conventional antibiotics. The most promising results were obtained with EME combined with ampicillin (AMP), which inhibited a greater number of strains compared to ciprofloxacin (CIP), effective only against *K. pneumoniae*. Specifically, the combination EME + AMP showed synergistic effects against *E. coli* NEWP0018 (β‐lactamase producer, FICI = 0.16) and *S. aureus* (clinical, β‐lactamase producer, FICI = 0.5), while EME + CIP synergized against *K. pneumoniae* (FICI = 0.19). The latter is particularly relevant as *K. pneumoniae* is on the WHO's critical priority list for resistance surveillance (WHO Bacterial Priority Pathogens List 2024). Additive interactions were also observed for the combination of EME + AMP, with FICI values of 0.516 for *S. pseudointermedius*, FICI of 0.53 for *E. coli* (clinical A, Shiga toxin‐producing), FICI of 0.75 for *Staphylococcus* sp., and FICI of 0.625 for ME + CIP against *A. baumannii*, indicating broader modulatory effects. These results suggest that EME possesses modulatory potential capable of enhancing antibiotic efficacy against resistant strains. Equally relevant results have already been described in the literature, with regard to the synergistic effect between natural products and antimicrobials so far used in therapy. *Pelargonium graveolens* essential oil and ciprofloxacin showed synergism against uropathogens *K. pneumoniae* KT2, *P. mirabilis* PRT3 (FICI = 0.375) and *S. aureus* ST 2 (FICI = 0.5) (Malik et al. 2011). Likewise, *Cannabis sativa* essential oil combined with ciprofloxacin synergized against *S. aureus*, *B. subtilis*, *E. coli*, and *K. pneumoniae* (FICI = 0.5) (Nafis et al. 2019). Triphala is an indigenous medicinal formulation used to treat a variety of diseases, like nose and skin wounds, tumors, peptic ulcer, cough and upper respiratory diseases, among others. It is composed of dried pericarp of the fruits of *Terminalia chebula* Retz. (Combretaceae), *Terminalia bellirica* (Gaertn.) Roxb. (Combretaceae) and *Emblica officinalis* L. (Euphorbiaceae) mixed in equal proportions (1:1:1). Triphala demonstrated synergistic activity with gentamicin against MDR Gram negative strains (*Serratia* species, *A. baumannii*, *K. penumoniae*, *Proteus* spp.) and with oxacillin against MRSA (Manoraj et al. 2019). A study conducted by Taukoorah et al. 2016 revealed the synergistic effect of the different extracts of *Piper betle* in combination with streptomycin or chloramphenicol against a wide range of bacterial strains. The highest activity was observed for the ethyl acetate extract–streptomycin combination against *E. coli*, *P. acnes*, and *S. epidermidis*. Compounds isolated from plants, in addition to extracts, have also demonstrated an effect on modulating antimicrobial activity. The alkaloid chanoclavine, isolated from *Ipomoea muricata*, could reduce the minimum inhibitory concentration (MIC) of tetracycline (TET) up to 16‐fold, by inhibiting the efflux pumps which seem to be ATPase‐dependent (Dwivedi et al. 2019). The monoterpene (‐)‐α‐pinene was investigated as a modulator of antimicrobial resistance in *Campylobacter jejuni*, and at least two different mechanisms were confirmed to contribute synergistically to its activity (Kovac et al. 2015). In lower concentrations it showed pronounced inhibition of antimicrobial efflux, and in higher concentrations it additionally targeted the membrane, with increased permeability, promoting the influx of antimicrobials. Together, these studies reinforce the potential of plant‐derived metabolites as antibiotic adjuvants.

Although further studies are warranted, including time–kill kinetic assays, which are the gold standard for evaluating *in vitro* synergy, previous studies have reported good agreement between checkerboard and time–kill results (Gaudereto et al. 2020; Nikolic et al. 2022; Bremmer et al. 2016; Soudeiha et al. 2017), supporting the checkerboard assay as a useful screening method.

Inhibition of bacterial biofilm formation represents a critical strategy in addressing antimicrobial resistance. Biofilms are defined as sessile microbial communities adhered to biotic or abiotic surfaces, embedded within a self‐produced extracellular polymeric matrix. Within this protective environment, bacteria exhibit pronounced tolerance to conventional antibiotics, host immune responses, and external stressors, which contribute to their persistence and therapeutic recalcitrance (Trentin et al. 2011; Woo et al. 2017; Parai et al. 2020). In this context, EME demonstrated notable antibiofilm activity, particularly at the concentration of 500 µg/mL, where it inhibited biofilm formation by *S. aureus* (72.3% ± 7.8) and *E. coli* (68.0% ± 3.6). The antibiofilm effect was more pronounced against Gram‐positive bacteria, with approximately 70% inhibition maintained even at the lowest concentration tested. Atomic force microscopy images of *S. aureus* biofilms formed in the presence of EME at the lowest concentration evaluated using the crystal violet assay (250 µg/mL), compared with the untreated control, supported these findings by revealing marked treatment‐induced alterations in the biofilm polymeric matrix.

It is estimated that biofilm‐associated microorganisms are implicated in more than 65% of human infections, including endocarditis, otitis media, prostatitis, periodontitis, conjunctivitis, vaginitis, and infections related to cystic fibrosis (Trentin et al. 2011; Woo et al. 2017; Parai et al. 2020; Qian et al. 2020; Sandasi et al. 2010). Moreover, biofilms are frequent colonizers of medical devices, such as catheters and prostheses, thereby playing a major role in the dissemination of pathogenic bacteria. Therefore, the ability of EME to impair biofilm formation further highlights its pharmacological potential. Stimulation of biofilm formation was observed in both bacterial strains at the highest concentration tested (1000 µg/mL). Previous studies have described a paradoxical response known as the “Eagle effect” in bacterial and fungal biofilms, in which higher antimicrobial concentrations exert weaker inhibitory effects than lower concentrations (Jacobson et al. 2024; Awad et al. 2025). The increased biofilm biomass observed following EME treatment may reflect a similar response; however, further studies are required to confirm the underlying mechanism.

Following LC–MS analysis of the bioactive extract, detailed compound annotation revealed a range of metabolites, including ellagic acid derivatives, triterpenes, and various flavonoids, mostly in glycosylated forms. Some compounds tentatively annotated in the chemical profile of EME have previously been reported to exhibit antimicrobial and biofilm‐inhibitory activities, such as asiatic acid (Garo et al. 2007). Other examples include terminolic acid and other pentacyclic triterpenes, which, individually or in specific combinations, have shown significant antibacterial effects against *Bacillus subtilis*, *Escherichia coli*, *Shigella sonnei*, and *Staphylococcus aureus (*Djoukeng et al. 2005; Chung et al. 2011). Therefore, these compounds may represent potential contributors to the observed activity of the *M. elliptica* extract against multidrug‐resistant bacteria. Beyond triterpenes, the flavonoids detected in EME may also be associated with the observed antimicrobial and synergistic effects. Quercetin, for example, has demonstrated significant synergistic activity in combination with antibiotics, particularly against multidrug‐resistant strains of *Pseudomonas aeruginosa (*Vipin et al. 2020 *)*. In their study, Vipin et al. (2020) reported that quercetin enhanced the efficacy of conventional antibiotics, such as levofloxacin, ceftriaxone, gentamicin, tobramycin, and amikacin, against *P. aeruginosa* strains isolated from catheter‐associated urinary tract infections. Synergistic interactions were confirmed by checkerboard and time‐kill assays, with combinations achieving over 80% inhibition of biofilm formation and biofilm cell viability. Furthermore, infection models using HEK‐293T cells showed a significant reduction in cytotoxic effects, suggesting a protective role for quercetin‐antibiotic combinations (Vipin et al. 2020).

In the present acute toxicity assay, oral administration of the ethanolic extract of *M. elliptica* (2000 mg/kg) did not induce overt signs of toxicity in mice. No significant alterations were observed in body weight gain, histomorphology, or in the relative weights of most organs, indicating the absence of systemic damage. Minor variations were detected, such as reduced liver weight in males and stomach weight in females, although both remained within physiological ranges. Hematological analysis revealed an increase in leukocyte counts in males, whereas biochemical analysis showed a reduction in bilirubin levels in both sexes.

These findings complement previous reports on *Mouriri pusa*, a related Cerrado species traditionally used to treat gastrointestinal disturbances, which demonstrated gastroprotective and regenerative actions on the gastric mucosa without evidence of toxicity (Vasconcelos et al. 2008). Similarly, *M. elliptica* has been reported to possess gastroprotective, ulcer‐healing, and anti‐*Helicobacter pylori* activities, attributed to phenolic acid derivatives, acylglycoflavonoids, and condensed tannins (Moleiro et al. 2009). The present results are consistent with these studies, further supporting the ethnopharmacological relevance of the genus *Mouriri* and suggesting a favorable safety profile for *M. elliptica* following acute exposure, although future chronic toxicity studies are recommended. The reduction in bilirubin levels and the modest decrease in liver weight may be associated with antioxidant properties previously described for *Mouriri* extracts, while the leukocytosis detected in males reinforces evidence of immunomodulatory activity possibly mediated by polyphenolic compounds (Tafere et al. 2020; Taiwo et al. 2009; Olusola et al. 2015; Doan et al. 2022).

Taken together, these data identify *M. elliptica* as a promising source of bioactive metabolites with antibacterial and antibiofilm potential, without evidence of acute toxicity under the conditions evaluated. Its activity against animal‐derived resistant strains underscores the relevance of this species within a One Health perspective, with implications for both public health and veterinary medicine. Further investigations, including sub‐chronic toxicity and mechanistic studies, are warranted to advance the development of *M. elliptica*‐based therapeutic strategies.

In addition, *M. elliptica* occurs naturally in the Cerrado phytogeographic domain and may therefore be considered an ecologically resilient species, adapted to seasonal water stress and recurrent fires. Previous studies have demonstrated its potential for cultivation and propagation (Assis et al. 2016; Vasconcelos et al. 2010b; Lima et al. 2016). Together with the production of edible fruits, these characteristics enhance its value as a potential source of antimicrobial agents, food, and income for local communities, supporting the sustainable management and socioeconomic valorization of regional biodiversity.

Brazil's vast biodiversity provides an unparalleled opportunity to identify novel natural products with antimicrobial activity. The promising results obtained for EME encourage further investigations in this field to develop alternative treatments against multidrug‐resistant bacteria, thereby addressing the emerging issue of antimicrobial resistance.

## 4 Materials and Methods

### 4.1 Collection of Plant Material

The leaves of *M. elliptica* were collected in the Cerrado biome of Mato Grosso do Sul, Brazil, on April 14, 2019, in the municipality of Campo Grande (MS) (lat: −20.966 long: −54.5525), and subsequently air‐dried at room temperature. The plant material was taxonomically identified by Prof. Dr. Flávio M. Alves (INBIO‐UFMS). A voucher specimen was deposited in the CGMS Herbarium under the registration number CGMS 73098. License for research on Brazil's biodiversity #A27C538, issued by National System for the Management of Genetic Heritage and Associated Traditional Knowledge (SISGEN/Brazil).

### 4.2 Preparation of the EME

The dried leaves of *M. elliptica* (1 kg) were ground in a knife mill to obtain a fine powder. The powdered material was subjected to exhaustive maceration with ethanol (plant‐to‐solvent ratio of approximately 1:10, w/v) at room temperature, with occasional manual agitation to improve solvent penetration. Every 24 h, the supernatant was collected and concentrated under reduced pressure, then stored in hermetically sealed amber glass bottles at –20°C. This procedure was repeated over a 7‐day period to yield 86.5 g of dried extract.

The resulting crude extract was transferred to amber glass vials and stored under refrigeration (4°C) and protected from light until further biological and chemical analyses.

### 4.3 HPLC‐DAD‐MS/MS Chemical Profiling

Aliquot of the ethanolic extract from the leaves of *M. elliptica* (EME) (10 µL of methanolic solution at 1.0 mg/mL) was injected into the HPLC‐DAD‐MS/MS. The analyzes were performed using an LC‐DAD‐HRESIMS system equipped with a SIL‐20A autosampler, a DGU‐20A3r vacuum degasser, a thermostated CTO‐20A column compartment, and an LC‐20AD pump, coupled to an SPD‐M20A DAD (all Shimadzu, Japan) and a micrOTOF Q‐III high‐resolution time‐of‐flight mass spectrometer (Bruker Daltonics, USA) with an electrospray ionization (ESI) ion source, operating in negative ion mode (120–1200 Da and collision energy 45–65 V). The eluent system used was water and methanol (both containing 0.1% formic acid), ranging from 3% to 80% of methanol (v/v), totaling 45 min of analysis, with a flow of 0.2 mL/min and positive ionization mode.

The annotation of the compounds presents in the chemical profile, as well as the proposition of the molecular formulas (considering only error values < 5 ppm), were performed through data analysis using the Compass DataAnalysis 4.2 software (Bruker, USA) and through a database assembled from SciFinder (Chemical Abstracts Service–CAS, USA) data platform, in which compounds already registered for the Melastomataceae family were searched. For data comparison, the RT, [M‐H]^+^ ions, and their main molecular ions, *m/z* values, fragmentation patterns, search in MS databases (GNPS), UV absorption spectra, and data obtained from isolated compounds and in the literature were analyzed.

### 4.4 Evaluation of Antimicrobial Activity

To evaluate antimicrobial activity, commercial and clinical isolated bacterial strains were used. The commercial ones were *Staphylococcus aureus* NEWP0023; *Escherichia coli* NEWP0022 and *E. coli* NEWP0018. Clinically isolated strains were: *S. aureus* resistant to penicillin G, erythromycin, and clarithromycin; *Staphylococcus* sp., resistant to amoxicillin, doxycycline, clindamycin, penicillin, oxacillin, norfloxacin, cefoxitin, azithromycin (from veterinary source); *E. coli* resistant to clindamycin, penicillin, and oxacillin (Shiga toxin‐producing *E. coli* A, from veterinary source); *E. coli* resistant to azithromycin, ampicillin, doxycycline, clindamycin, penicillin, oxacillin, norfloxacin, and cefoxitin (*E. coli* B, from veterinary source); *Staphylococcus pseudointermedius* resistant to amoxicillin + clavulanic acid, gentamicin, neomycin, azithromycin, cephalexin, cephalothin, streptomycin, marbofloxacin (from veterinary source); *Acinetobacter baumannii* resistant to amikacin, ciprofloxacin, gentamicin, imipenem, levofloxacin, and meropenem and *Klebsiella pneumoniae* resistant to amoxicillin‐clavulanate, ampicillin, aztreonam, cefepime, cefoxitin, ceftriaxone, cefuroxime, ciprofloxacin, colistin, ertapenem, gentamicin, imipenem, levofloxacin, meropenem, piperacillin‐tazobactam, intermediate to tigecycline.

Human clinical strains were provided (identified and characterized) by the Clinical Analysis Center of the University Hospital, and animal‐origin strains by the Veterinary Hospital, both at the Federal University of Mato Grosso do Sul (Campo Grande, Brazil). The antimicrobial activity of the extract alone was determined by the broth microdilution method, as described by Manda et al. (2018). All experiments were performed in triplicate. Triphenyl tetrazolium chloride (TTC) was used as colorimetric indicator, and the minimum inhibitory concentration (MIC) was visually defined as the lowest concentration of each sample where no color change (colorless to red) occurred and was expressed in μg/mL.

Synergistic interactions were evaluated using the checkerboard microtiter assay method, as described by Solarte et al. (2017). The MIC of the combinations was evaluated, and the fractional inhibitory concentration (FIC) and fractional inhibitory concentration index (FICI) were calculated using the formulas: FIC = combined MIC of extract or antibiotic/individual MIC of extract or antibiotic FICI = FIC of extract + FIC of antibiotic. The FICI value will be interpreted as: synergistic (FICI ≤ 0.5), additive (0.5 < FICI ≤ 1), indifferent (1 < FICI ≤ 4), and antagonistic (FICI > 4) (Simões et al. 2009; Mbaveng et al. 2015; Solarte et al. 2017; Lim et al. 2016; Ahumada‐Santos et al. 2016).

### 4.5 Evaluation of Antibiofilm Activity

To evaluate antibiofilm activity, the commercial strains *Staphylococcus aureus* NEWP0023 and *Escherichia coli* NEWP0022 were used. The inhibition of biofilm formation was evaluated employing crystal violet, using the method described by Trentin el al. 2011 (Trentin et al. 2011), with some modifications. The samples were prepared using sterile distilled water and DMSO, to achieve a final maximum DMSO concentration of 2%. Serial dilutions were performed in 96‐well plates prepared with Mueller‐Hinton broth to achieve a final concentration of 250 to 1000 μg/mL, with a final volume of 50 μL in each well. A bacterial suspension in Mueller‐Hinton broth (50 μL, 0.5 McFarland) was added and the plates were incubated again at 36°C for 18 h. Experiments were conducted in triplicate. The content of the wells was removed and the wells were washed three times with sterile saline solution. The remaining attached bacteria were heat‐fixed at 60°C for 1 h and the adherent biofilm layer formed was stained with 0.4% crystal violet for 15 min at room temperature. After washing the plates with sterile water, the bound crystal violet was dissolved with 200 μL of 95% ethanol and absorbance was measured at 570 nm (EZ Read 400, Biochrom). The biofilm formation control was considered to represent 100% of biofilm formation, and the extract was replaced by 50 μL of the solvent. Values higher than 100% represent a stimulation of biofilm formation in comparison to the control.

### 4.6 Atomic Force Microscopy (AFM) Analysis of Biofilm

To investigate *S. aureus* biofilm structure, the test for inhibition of its formation was performed using 12‐well plates. Sterile glass slides (20 ×20 mm, 0.13–0.16 mm thickness, Olen, Brazil) were placed inside wells prepared with 1.5 mL of Mueller Hinton broth and 1.5 mL of a solution of the extract in water + 1% DMSO, to reach a final concentration of 250 μg/mL in each well. For the blank, the extract solution was replaced by 1.5 mL of water + 1% DMSO. Wells were inoculated with 150 μL of a 0.5 McFarland bacterial solution in sterile saline, prepared from a 24h‐culture in Mueller‐Hinton agar. The plates were incubated at 36°C for 24 h, the glass slides were washed with sterile water and heat‐fixed at 60°C for 1 h and then analyzed via AFM to visualize the three‐dimensional structure of the biofilm. Topography maps were recorded on a Park XE 70 microscope (Park Systems, Suwon–Korea) equipped with SmartScan software version 1.0 Build 83. The measurements were conducted using a PPP‐NCHR 10 M probe with a nominal resonance frequency of 341.3 kHz and 40 Nm^−1^ force constant. All measurements were made at room temperature (25°C ± 5°C) and relative humidity (20% ± 10%). Images were treated using XEI software (Park Systems, Suwon‐Korea) version 1.8.1 Build 214.

### 4.7 *In Vivo* toxicity

#### 4.7.1 Animals and Ethical Approval

Male and female Swiss mice (*Mus musculus*; 4–6 weeks; 18–25 g) were obtained from the Central Animal Facility (Biotério Central) of the Federal University of Mato Grosso do Sul (UFMS, Brazil). Animals were housed (4–5 per cage) under controlled conditions (22°C ± 2°C; 50%–60% relative humidity; 12 h light/12 h dark photoperiod) with standard chow and water *ad libitum*. After a 7‐day acclimatization, mice were randomly assigned to groups.

All procedures complied with Brazilian legislation (Law No. 11.794/2008 and Decree No. 6.899/2009) and CONCEA guidelines, followed the ARRIVE recommendations, and were approved by the UFMS Animal Ethics Committee (CEUA) [protocol No. 1.146/2020]. The authorization covered a total of 36 Swiss mice (18 females and 18 males) sourced from the UFMS Central Animal Facility. Experiments reported here were conducted within this authorization window. For the present acute toxicity experiment with EME, group sizes were *n* = 5/sex/group for hematology and relative organ weights, and a subset of *n* = 3/sex/group was used for serum biochemistry.

### 4.8 Experimental Design and Dosing (Acute Oral Toxicity)

The acute oral toxicity assay followed the OECD “limit test” concept (TG 423) with adaptations. Within each sex, mice received either vehicle (distilled water, 10 mL/kg; control) or EME (single oral dose, 2000 mg/kg) by oral gavage (stainless‐steel gavage needle) after a short fasting period (3–4 h; water available). The observation period was 14 days post‐dose.

### 4.9 Clinical Observations and Body Weight

Animals were observed for mortality and clinical signs at 0.5, 1, 2 and 4 h after dosing and at least once daily thereafter for 14 days (piloerection, posture, locomotion, tremors/convulsions, salivation, respiration, cyanosis, grooming, reactivity, fecal/urinary output). Body weight was recorded on days 0 (pre‐dose), 7 and 14.

### 4.10 Blood Collection and Hematology

On day 14, mice were anesthetized with Xylazine 2%, Syntec; Ketamyne 10%, Syntec; Xilazine:Ketamyne, 1:2, and blood was collected via retro‐orbital plexus. Samples for hematology were transferred to EDTA tubes and analyzed in an automated analyzer for WBC (×10^3^/µL), RBC (×10^6^/µL), HGB (g/dL), HCT (%), MCV (fL), MCH (pg), MCHC (g/dL), and PLT (×10^3^/µL).

### 4.11 Serum Biochemistry

Blood for biochemistry was collected, allowed to coagulate, and centrifuged (1500–2000 g, 10 min, room temperature). Serum was analyzed for glucose (g/dL), albumin (g/dL), total bilirubin (mg/dL), ALT (U/L), and AST (U/L) using standard enzymatic/colorimetric kits.

### 4.12 Necropsy and Relative Organ Weights

Immediately after blood collection, animals were euthanized under deep anesthesia by exsanguination. A gross necropsy was performed. Organs were excised, blotted, and weighed individually: liver, kidneys, spleen, heart, lungs, stomach, pancreas, and reproductive organs. Relative organ weight (ROW) was calculated as *(organ weight/terminal body weight) × 100*.

### 4.13 Histopathology

Organs were fixed in 10% neutral‐buffered formalin (≥ 24 h), processed routinely, embedded in paraffin, sectioned at 4–5 µm, and stained with hematoxylin–eosin (H&E). Slides were examined under light microscopy (100×) to assess white pulp (WP) and red pulp (RP) architecture and to detect inflammatory or degenerative changes. Representative photomicrographs were acquired using identical exposure settings.

### 4.14 Data Presentation and Statistical Analysis

Data are expressed as mean ± SEM. Normality and homogeneity of variance were assessed by Shapiro–Wilk and Levene tests. Body weight over time: two‐way ANOVA (treatment × time; sex‐stratified), followed by Bonferroni post hoc test. Relative organ weights, hematology and biochemistry: one‐way ANOVA within each sex (Control vs. EME) with Bonferroni post hoc test. A significance level of *p* < 0.05 was adopted. Analyses were performed in GraphPad Prism (version 5, GraphPad Software, USA).

## Briefs

Ethanolic extract of *Mouriri elliptica* exhibits antimicrobial, synergistic, and antibiofilm activities against clinical multidrug‐resistant bacteria, without acute toxicity in vivo.

## Synopsis

The ethanolic extract of *Mouriri elliptica* leaves demonstrated significant antibacterial effects, including strong synergism with conventional antibiotics against clinical multidrug‐resistant strains, in addition to inhibition of biofilm formation. LC‐MS profiling revealed 41 metabolites, and acute toxicity assays confirmed its safety in vivo. These findings highlight the potential of *M. elliptica* as a natural adjuvant to enhance antibiotic efficacy against bacterial resistance.

## Author Contributions

**Talita Vilalva Freire:** visualization, writing – original draft, data curation. **Ana Carolina de Freitas Marques:** investigation, formal analysis. **Valéria dos Santos Gonçalves:** investigation, methodology. **Iluska Senna Bonfá Moslaves:** investigation, visualization. **Mônica Cristina Toffoli‐Kadri:** investigation, methodology. **Arthur Feliciano Camargo da Silva:** investigation, methodology. **Martha Janete Giz:** investigation, methodology, writing – review and editing. **Patrícia de Oliveira Figueiredo:** visualization, data curation. **Ana Camila Micheletti:** investigation, methodology, formal analysis, writing – original draft, validation. **Nídia Cristiane Yoshida:** conceptualization, investigation, formal analysis, writing – original draft, visualization, data curation, supervision, methodology, writing – review and editing, funding acquisition, project administration.

## Ethics Statement

The authors have nothing to report.

## Supporting information


Supporting File

## Data Availability

The data that support the findings of this study are available on request from the corresponding author. The data are not publicly available due to privacy or ethical restrictions.
