# Supplementary material for: Adjuvant Antibacterial Effects of Mouriri Elliptica Against Clinical Multidrug Resistant Gram‐Negative Bacterial Strains
Source: Microbiologyopen. 2026 Sep 9;15(5):e70392. doi: 10.1002/mbo3.70392 (PMC13558664; doi:10.1002/mbo3.70392)
Supplement: Supplementary file 1 [file MBO3-15-e70392-s001.docx]

Supplementary Material

ANTIBACTERIAL EFFECTS OF *MOURIRI ELLIPTICA* AGAINST CLINICAL MULTIDRUG RESISTANT GRAM-NEGATIVE BACTERIAL STRAINS

*Talita Vilalva Freire^1^, Ana Carolina de Freitas Marques^2^, Valéria dos Santos Gonçalves^1^, Iluska Senna Bonfá Moslaves^3^, Mônica Cristina Toffoli-Kadri^3^, Arthur Feliciano Camargo da Silva^4^, Martha Janete Giz^4^_,_ Patrícia de Oliveira Figueiredo^1^, Ana Camila Micheletti^1*^, Nídia Cristiane Yoshida^1*^*

^1^Federal University of Mato Grosso do Sul, Laboratory of Research on Bioactive Natural Products (PRONABio), Institute of Chemistry (INQUI), Campo Grande - MS, Brazil.

^2^Federal University of Mato Grosso do Sul, Institute of Biology (INBIO), Campo Grande - MS, Brazil.

^3^Federal University of Mato Grosso do Sul, Faculty of Pharmaceutical Sciences (FACFAN), Campo Grande - MS, Brazil

^4^Federal University of Mato Grosso do Sul, Laboratory of Electrochemistry of Advanced Materials (LEMA), Institute of Chemistry (INQUI), Campo Grande - MS, Brazil.

**Table S1.** Effect of the ethanolic extract of *Mouriri elliptica* (EME) leaves on body weight gain over a 14-day period.

**Table S2.** Organ weights of female and male mice after a 14-day treatment period with the ethanolic extract of *Mouriri elliptica* (EME) leaves.

**Table S3.** Effect of treatment with the ethanolic extract of *Mouriri elliptica* (EME) on hematological parameters of mice following the acute toxicity assay.

**Table S4.** Effect of the ethanolic extract of *Mouriri elliptica* (EME) on the biochemical parameters of mice following the acute toxicity assay.

**Table S1.** Effect of the ethanolic extract of *Mouriri elliptica* (EME) leaves on body weight gain over a 14-day period.

|  | **Female** | | **Male** | |
| --- | --- | --- | --- | --- |
| **Day** | **Control** | **EME** | **Control** | **EME** |
| 1 | 19.373±1.715 | 17.722±0.584 | 23.177±1.001 | 19.888±0.660 |
| 7 | 21.843±0.571 | 22.964±0.674 | 28.027±1.851 | 26.886±1.202^a^ |
| 14 | 23.900±0.780 | 24.384±0.532 | 29.670±3.118 | 27.570±1.090^b^ |

Results are expressed as mean ± SEM (Standard Error of the Mean), n = 5/group. EME = *Mouriri elliptica* ethanolic extract. Animals received either water (10 mL/kg, control group) or the extracts orally as a single dose (2000 mg/kg). Body weight of females and males was recorded weekly over a 14-day period. Two-way ANOVA followed by Bonferroni’s test, n = 5/group, p < 0.05. a = Difference compared with EME females on day 7 of treatment; b = Difference compared with EME females on day 14 of treatment.

**Table S2.** Organ weights of female and male mice after a 14-day treatment period with the ethanolic extract of *Mouriri elliptica* (EME) leaves.

| **Organ** | **Female** | | **Male** | |
| --- | --- | --- | --- | --- |
|  | **Control** | **EME** | **Control** | **EME** |
| Kidney | 0.177 ± 0.010 | 0.162 ± 0.006 | 0.234 ± 0.029 | 0.222 ± 0.011 |
| Liver | 1.383 ± 0.151 | 1.231 ± 0.043 | 1.644 ± 0.105 | 1.487 ± 0.085 * |
| Spleen | 0.125 ± 0.013 | 0.116 ± 0.006 | 0.114 ± 0.008 | 0.101 ± 0.006 |
| Heart | 0.150 ± 0.001 | 0.165 ± 0.008 | 0.159 ± 0.012 | 0.156 ± 0.007 |
| Lung | 0.232 ± 0.051 | 0.193 ± 0.009 | 0.234 ± 0.025 | 0.211 ± 0.005 |
| Stomach | 0.964 ± 0.322 | 0.595 ± 0.022 ** | 0.441 ± 0.039 | 0.486 ± 0.023 |
| Pancreas | 0.092 ± 0.028 | 0.114 ± 0.005 | 0.117 ± 0.015 | 0.116 ± 0.009 |
| Reproductive | 0.181 ± 0.038 | 0.221 ± 0.024 | 0.252 ± 0.036 | 0.273 ± 0.018 |

**Results are expressed as mean ± SEM, n = 5/group. EME = *Mouriri elliptica* ethanolic extract. Animals received either water (10 mL/kg, control group) or the extract orally as a single dose (2000 mg/kg). Organ weights were recorded at the end of the 14-day period. One-way ANOVA followed by Bonferroni’s test, n = 5/group. *p < 0.05; **p < 0.01; compared with control.

**Table S3.** Effect of treatment with the ethanolic extract of *Mouriri elliptica* (EME) on hematological parameters of mice following the acute toxicity assay.

| **Parameter** | **Females** | | **Males** | |
| --- | --- | --- | --- | --- |
|  | **Control** | **EME** | **Control** | **EME** |
| WBC (×10³/µL) | 8.23 ± 0.491 | 10.28 ± 1.224 | 12.93 ± 1.676 | 18.76 ± 1.289 * |
| RBC (×10⁶/µL) | 10.00 ± 0.000 | 9.86 ± 0.040 | 9.43 ± 0.371 | 10.10 ± 0.352 |
| HGB (g/dL) | 16.27 ± 0.145 | 16.28 ± 0.058 | 15.33 ± 0.330 | 16.22 ± 0.448 |
| HCT (%) | 56.90 ± 0.173 | 55.60 ± 0.316 | 53.23 ± 1.670 | 57.10 ± 1.904 |
| MCV (fL) | 55.97 ± 1.157 | 54.78 ± 1.681 | 56.47 ± 1.378 | 56.64 ± 0.631 |
| MCH (pg) | 17.37 ± 1.081 | 16.16 ± 0.344 | 16.30 ± 0.404 | 16.10 ± 0.207 |
| MCHC (g/dL) | 31.17 ± 2.621 | 29.58 ± 0.322 | 28.83 ± 0.418 | 28.42 ± 0.309 |
| PLT (×10³/µL) | 1050.0 ± 72.0 | 937.2 ± 123.0 | 1436.0 ± 145.0 | 972.6 ± 76.6 |

**Results are expressed as mean ± SEM, n = 5/group. EME = *Mouriri elliptica* ethanolic extract. WBC = white blood cells; RBC = red blood cells; HGB = hemoglobin; HCT = hematocrit; MCV = mean corpuscular volume; MCH = mean corpuscular hemoglobin; MCHC = mean corpuscular hemoglobin concentration; PLT = platelets. Animals received either water (control group) or the extract orally as a single dose (2000 mg/kg). Hematological parameters of female and male mice were recorded at the end of a 14-day period. ANOVA followed by Bonferroni’s test, n = 5/group. *p < 0.05; compared with control.

**Table S4.** Effect of the ethanolic extract of *Mouriri elliptica* (EME) on the biochemical parameters of mice following the acute toxicity assay.

|  | **Females** | | **Males** | |
| --- | --- | --- | --- | --- |
| **Parameter** | **Control/Water**  **(10 mL/kg)** | **EME**  **(2000 mg/kg)** | **Control/Water**  **(10 mL/kg)** | **EME**  **(2000 mg/kg)** |
| Glucose (g/dL) | 77.20 ± 17.85 | 123.00 ± 0.87 | 77.20 ± 17.85 | 136.00 ± 0.00 |
| Albumin (g/dL) | 2.52 ± 0.10 | 2.45 ± 0.15 | 2.98 ± 0.31 | 2.60 ± 0.08 |
| Bilirubin (mg/dL) | 0.60 ± 0.03 | 0.24 ± 0.00 * | 0.60 ± 0.03 | 0.38 ± 0.00 *** |
| ALT (U/L) | 4.40 ± 2.09 | 22.00 ± 3.46 | 13.96 ± 1.04 | 7.00 ± 1.73 |
| AST (U/L) | 31.00 ± 5.20 | 34.00 ± 3.79 | 25.00 ± 3.21 | 37.00 ± 5.51 |

**EME = *Mouriri elliptica* ethanolic extract. Animals received either water (control group) or the extract orally as a single dose. Biochemical parameters of female and male mice were recorded at the end of a 14-day period. Results are expressed as mean ± SEM, n = 3/group. ANOVA followed by Bonferroni’s test, n = 3/group. *p < 0.05; *p < 0.001, compared with control
